# Haplotype-resolved genome assembly provides insights into evolutionary history of the *Actinidia arguta* tetraploid

**DOI:** 10.1186/s43897-024-00083-6

**Published:** 2024-02-06

**Authors:** Feng Zhang, Yingzhen Wang, Yunzhi Lin, Hongtao Wang, Ying Wu, Wangmei Ren, Lihuan Wang, Ying Yang, Pengpeng Zheng, Songhu Wang, Junyang Yue, Yongsheng Liu

**Affiliations:** 1 School of Horticulture, Anhui Agricultural University, Hefei, 230036 China; 2 School of Forestry Science and Technology, Lishui Vocational and Technical College, Lishui, 323000 China; 3 Ministry of Education Key Laboratory for Bio-Resource and Eco-Environment, College of Life Science, State Key Laboratory of Hydraulics and Mountain River Engineering, Sichuan University, Chengdu, 610064 China

**Keywords:** *Actinidia arguta*, Genomics, Polyploid, Haplotype, Stress tolerance

## Abstract

*Actinidia arguta*, known as hardy kiwifruit, is a widely cultivated species with distinct botanical characteristics such as small and smooth-fruited, rich in beneficial nutrients, rapid softening and tolerant to extremely low temperatures. It contains the most diverse ploidy types, including diploid, tetraploid, hexaploid, octoploid, and decaploid. Here we report a haplotype-resolved tetraploid genome (*A. arguta* cv. ‘Longcheng No.2’) containing four haplotypes, each with 40,859, 41,377, 39,833 and 39,222 protein-coding genes. We described the phased genome structure, synteny, and evolutionary analyses to identify and date possible WGD events. *K*_*s*_ calculations for both allelic and paralogous genes pairs throughout the assembled haplotypic individuals showed its tetraploidization is estimated to have formed ~ 1.03 Mya following A*d-α* event occurred ~ 18.7 Mya. Detailed annotations of NBS-LRRs or CBFs highlight the importance of genetic variations coming about after polyploidization in underpinning ability of immune responses or environmental adaptability. WGCNA analysis of postharvest quality indicators in combination with transcriptome revealed several transcription factors were involved in regulating ripening kiwi berry texture. Taking together, the assembly of an *A. arguta* tetraploid genome provides valuable resources in deciphering complex genome structure and facilitating functional genomics studies and genetic improvement for kiwifruit and other crops.

**Graphical Abstract:**

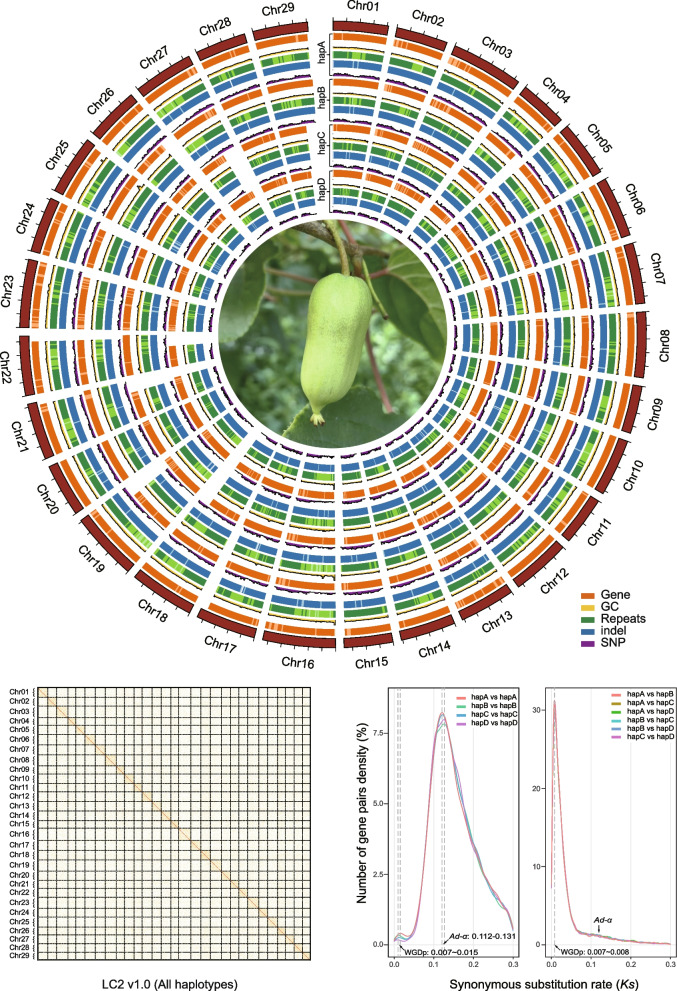

**Supplementary Information:**

The online version contains supplementary material available at 10.1186/s43897-024-00083-6.

## Core

For the first time we report a haplotype-resolved tetraploid genome of *A. arguta* containing the phased genome structure, synteny, and evolutionary history of the tetraploidization and date the possible WGD events. Comprehensive annotations of NBS-LRRs or CBFs emphasize the importance of genetic variations occurring after tetraploidization in underpinning ability of immune responses or environmental adaptability. This study sheds light to understand complex genome evolution that will promote functional genomics studies and genetic improvement for kiwifruit and other crops.

## Gene and accession numbers

The raw Hi-Fi, Hi-C and RNA-seq reads, assembled genome and annotations generated in this study have been deposited in the NGDC database (https://ngdc.cncb.ac.cn/) with the accession number PRJCA022944.

## Introduction

Actinidiaceae is the basal family within Ericales, consisting of the genera *Actinidia*, *Saurauia* and *Clematoclethra* (Dickison et al. 1982). In *Actinidia* (commonly known as kiwifruit), approximately 54 species and 75 taxonomic groups have been described (Li et al. 2007). All members of the genus are perennial, deciduous and climbing plants and are functionally dioecious. The fruits are berries with seeds embedded in a fleshy pericarp. The kiwifruit for commercial cultivation includes *A. chinensis* Planchon, *A. deliciosa* (*A. chinensis* var. deliciosa A. Chevalier), *A. kolomikta* (Maxim. et Rupr.) Maxim, *A. arguta* (Siebold and Zuccarini) Planchon ex Miquel and *A. eriantha* Bentham. The kiwifruit taxa are usually reticular polyploids with a basic chromosome number of x = 29 (McNeilage and Considine 1989).

**Fig. 1:**
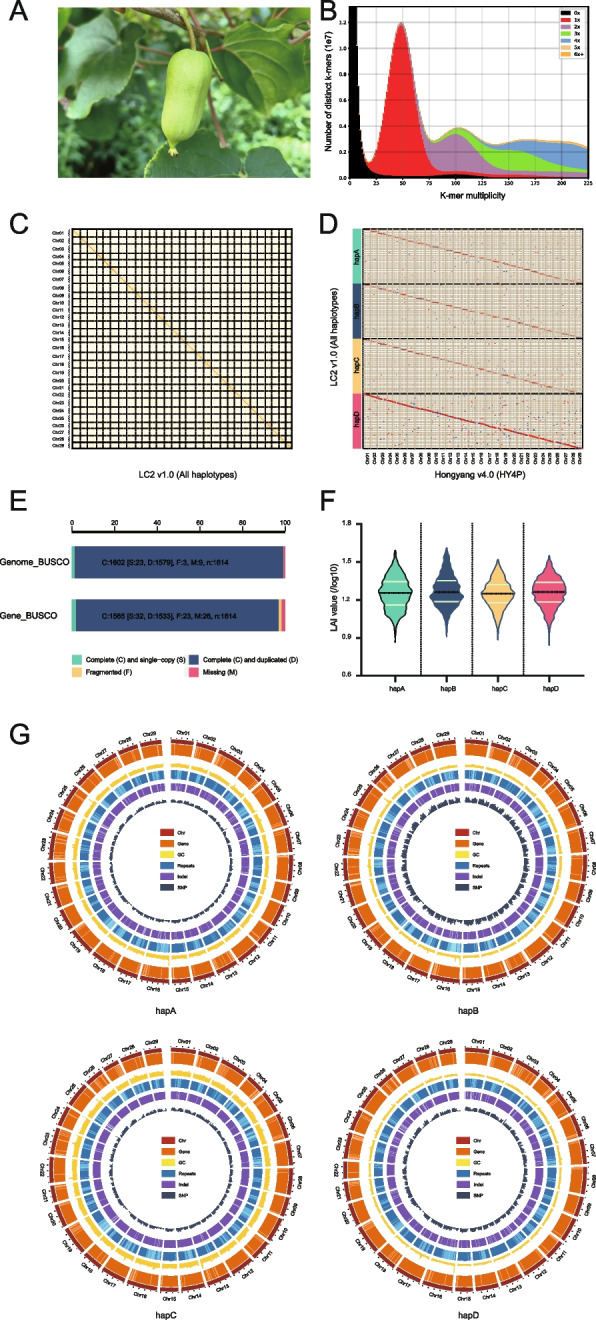
Quality assessment of the LC2 v1.0 genome assembly. **A** The plant with fruit of *A. arguta* cv. ‘Longcheng No.2’. **B** Comparison of the amount of distinct K-mers absent and copy number variation between LC2 v1.0 assembly and raw HiFi reads. The plots are colored to illustrate the times of specific K-mers referred from the reads appeared in the assembly. The blackness represents K-mers missing from the assembly, while color of red, purple, green, blue, yellow or orange represents K-mers that appear 1, 2, 3, 4, 5 or ≥ 6 times in the assembly, respectively. **C** Heatmap displaying Hi-C interacting signals of LC2 v1.0 chromosomes. Each homologous group contains four chromosomes. **D** Collinearity between the four haplotypes of LC2 v1.0 and HY4P. HY4P was used for adjusting the orientation of LC2 v1.0 chromosomes. **E** Genome BUSCO and gene BUSCO assessments exhibiting proportions classified as categories of complete and single-copy (S, green), complete and duplicated (D, blue), fragmented (F, yellow), and missing (M, pink). **F** Distribution of LAI scores among the assemblies of four haplotypes of LC2 v1.0. **G** Genomic features of the four haplotypes of LC2 v1.0. Tracks from outside to inside are chromosome identifiers (Chr), gene density (Gene), guanine-cytosine (GC) content, repeat density (Repeats), InDel density (InDel) and SNP density (SNP). All statistics were computed for windows of 500 Kb

*A. arguta* is also known as hardy kiwifruit, baby kiwi or kiwi berry due to its strong freezing tolerance and small-fruited botanical characteristics. The fruit is usually oval, nearly spherical, grape-sized, smooth and hairless, and can be eaten without peeling (Fig. 1A). Fruit of *A. arguta* is rich in nutrients, such as ascorbic acid, lutein, phenolics, and minerals, especially phosphorus, calcium, iron, and zinc (Krupa et al. 2011). In addition, it also possesses nutritional or medical effects, such as anti-inflammatory, antioxidant, cancer prevention and lowering blood pressure (Xu et al. 2021). The distribution areas of its wild resources include China, Japan, Russia, and the Korean Peninsula. In China, provinces such as Shandong, Liaoning, Jilin, and Heilongjiang are particularly rich in wild resources. In the late nineteenth century, kiwi berry was introduced from Japan to the United States, and commercial cultivation began in the latter half of the twentieth century. In 1955, kiwi berry was introduced to New Zealand and began commercial cultivation in the 1980s (Zhang et al. 2017a). China started the kiwi berry breeding and cultivation since the 1960s of the last century (Lu et al. 2020). Up to now, dozens of elite varieties of kiwi berry have been developed and commercially grown in Northeast Asia, Northern Europe, North America, and other high-latitude countries (Latocha 2017).

In recent years, increasing investigations have been dedicated to unraveling the underlying mechanisms of cold tolerance (Lin et al. 2021; Sun et al. 2021), canker resistance (Wang et al. 2020), phytonutrients metabolism or fruit quality (Lin et al. 2022) and gender (Akagi et al. 2023) using *A. arguta* germplasms, with a goal of genetic improvement through molecular breeding approaches. However, the progress of functional genomics studies has been relatively slow due to the lack of a reference genome. Since the first kiwifruit genome was released in 2013 (Huang et al. 2013), the reference genome of several *Actinidia* species such as *A. chinensis* (Pilkington et al. 2018; Wu et al. 2019; Yue et al. 2023), *A. chinensis* var. *deliciosa* (Xia et al. 2023)*, A. eriantha* (Tang et al. 2019; Wang et al. 2023; Yao et al. 2022), *A. arguta* (Akagi et al. 2023) and *A. latifolia* (Han et al. 2023) have been characterized, which laid a solid foundation for the development of functional genomics. The *Actinidia* genus manifests a variety of natural ploidy variations, with diploid (2n = 2x = 58), tetraploid (2n = 4x = 116), and hexaploid (2n = 6x = 174) being the most common (Huang et al. 1997). Among them, *A. chinensis* constitutes both diploid and tetraploid forms, while *A. deliciosa* is present primarily as hexaploid with sporadically distributed diploid accessions and entirely *A. eriantha* consists of only diploid individuals. By contrast, ploidy variation in *A. arguta* is much more complex, including diploid, tetraploid, hexaploid, octoploid, and decaploid, but majority of the cultivated varieties are tetraploids probably due to the advantageous growth vigor and/or abiotic/biotic stress resistance (Zhang et al. 2017b). Unfortunately, previously reported *Actinidia* genomes are all derived from diploid species, which prompts us to assemble a high-quality reference genome for *A. arguta* tetraploid. Actually, several plant polyploidy genomes have been assembled, such as sugarcane (Zhang et al. 2018), chrysanthemum (Song et al. 2023), and strawberry (Edger et al. 2019). Fortunately, recent advancements in sequencing technologies allow us to decipher the tetraploid *A. arguta* genome through a high-quality assembly.

In the present study, by integrating the HiFi and Hi-C sequencing technologies we assembled and characterized the genome of an *A. arguta* cultivar ‘Longcheng No.2’, a tetraploid individual putatively harboring four sets of chromosomes (2n = 4x = 116). Through comparative genomics studies, transcriptome sequencing and WGCNA analysis, we annotated candidate functional genes involved in fruit softening and texture formation, disease resistance and stress tolerance. We also present evidence of molecular evolution following the most recent whole genome duplication (A*d-α*) that accompanied autotetraploid formation ~ 1.03 million years ago. Our study provides valuable resources for genetic improvement and towards understanding the complex genome evolution and molecular mechanisms underlying the extensive adaption to diverse environments in *A. arguta*.

## Results

### Haplotype resolved assembly of a tetraploid *A*. *arguta* genome

Flow cytometry was used to confirm that *Actinidia arguta* cv. ‘Longcheng No.2’ is actually a tetraploid (Figure S1). To obtain high-quality genome assemblies, the genomic DNA of *A. arguta* cv. ‘Longcheng No.2’ (Fig. 1A) was sequenced and a total of 66.6 Gb (∼23.4 × coverage) of HiFi reads were generated using PacBio Sequel II (Table S1). The average length of the HiFi reads was > 16.3 kb (Table S1). In addition, chromosome conformation capture sequencing (Hi-C) libraries were constructed and sequenced using DNBSEQ-T7 (BGI) platform, generating 102 Gb (∼36.0 × coverage) clean reads for downstream grouping, ordering, orientation and verification of assembled unitigs (Table 1 and Table S2). A total of 1,104,265 unitigs sized 2919 Mb with an N50 of 0.94 Mb were assembled by hifiasm (Cheng et al. 2021) using the PacBio HiFi and Hi-C data (Table 1). Subsequently, these unitigs were phased and assembled following the ALLHiC pipeline with minor adjustments, generating a haplotype-resolved genome, namely LC2 v1.0 (Table S3).

**Table 1:** Summary statistics of *A. arguta* genome assemblies

**Genomic feature**	**hapA**	**hapB**	**hapC**	**hapD**
Total size of assembled unitigs (Mb)	2,919.0			
Number of unitigs	1,104,265			
N50 value of unitig length (Mb)	0.94			
Total size of assembled genomes (Mb)	615.2	595.0	570.2	552.7
Number of base chromosomes	29	29	29	29
Number of telomeres (pairs)	38 (12)	38 (13)	33 (11)	33 (9)
Number of definite centromeres	29	29	29	29
Genome BUSCOs (%)	99.2			
TE size (%)	41.02	39.51	38.85	38.89
GC content (%)	35.45	35.43	35.41	35.49
LTR assembly index score	16.86	17.62	16.93	17.15
Gene BUSCOs (%)	97.0			
Number of genes/transcripts	42,263/52,105	41,377/51,041	39,833/49,271	39,222/48,363
Number of shared genes	40,859	40,063	38,638	38,098
Number of specific genes	1,404	1,314	1,195	1,124

Four haplotypes were assembled in LC2 v1.0, with the primary haplotype (hereafter named hapA) and three alternate haplotypes (hereafter named hapB, hapC, hapD) individually containing 29 chromosomes with a total length of 615.2 Mb, 595.0 Mb, 570.2 Mb and 552.7 Mb, respectively (Table 1 and Table S3).

Subsequently, the accuracy of the four assembled haplotypes was assessed using a variety of approaches. Firstly, the spectra-graph plotted by the KAT program (Mapleson et al. 2017) unambiguously demonstrated that ‘Longcheng No.2’ is a typical tetraploid and the phasing of the assembled haplotypes is largely correct (Fig. 1B and Figure S1B). Secondly, Hi-C interaction matrices displayed a diagonal pattern that favors intra-chromosomal interactions in all chromosomes, indicating the high accuracy of phasing, ordering and orientation (Fig. 1C). Thirdly, genome completeness was evaluated by mapping various raw reads against the genome, and each of them showed high mapping rate, such as raw PacBio HiFi reads (> 99%), Hi-C reads (> 99%), or RNA-seq reads (> 97%) (Table S4). Collinearity between the four haplotypes of LC2 v1.0 and HY4P revealed consistency of the sequence orders (Fig. 1D). The quality of the assembly evaluated using BUSCO (Manni et al. 2021) showed a 99.2% completeness of the embryophyta_odb10 gene set in four haplotypes (Fig. 1E and Table 1). Consequently, we obtained a tetraploid *A. arguta* genome encompassing 116 chromosomes phased into four haplotypes, with 682, 749, 742, and 764 gaps, respectively (Table 1 and Table S3). Finally, long terminal repeat (LTR) annotation showed that the LTR assembly index (LAI) values for hapA, hapB, hapC and hapD were 16.86, 17.62, 16.93 and 17.15, respectively (Fig. 1F and Table S5), indicating that the quality of the assembly is up to the reference level (Ou et al. 2018).

Next, a total of 42,263, 41,377, 39,833 and 39,222 protein-coding genes in hapA, hapB, hapC and hapD were identified, respectively, capturing 97.0% of the embryophyta_odb10 BUSCO gene set (Table 1). Meanwhile, putatively 52,105, 51,041, 49,271 and 48,363 transcripts were predicted with an average of 1.23, 1.23, 1.24 and 1.23 splice variants from the entire genes’ pools of hapA, hapB, hapC and hapD (Fig. 1G and Table 1). Out of these protein-coding genes, 36,575 (86.54%), 36,053 (87.13%), 34,785 (87.33%), 34,336 (87.54%) were functionally annotated in a comprehensive database of eggNOG-mapper (Cantalapiedra et al. 2021), respectively.

### Relatively conserved potential candidates of telomeres and centromeres

**Fig. 2:**
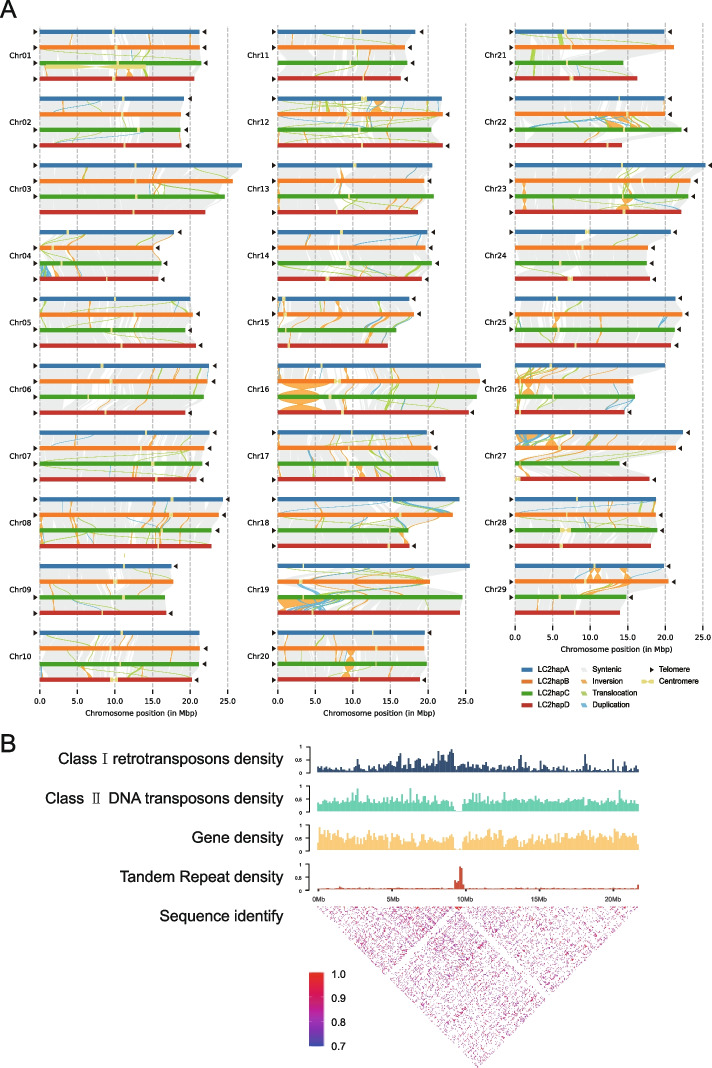
Structure validation of LC2 v1.0 genome. **A** Structure of the haplotype-resolved chromosomes in LC2 v1.0. All 116 chromosomes of four haplotypes are drawn to scale and the ruler indicates chromosome length. Collinearity between haplotypes with syntenic regions is shown as gray lines, inversions as orange lines, translocations as green lines, and duplications as blue lines. Black triangles indicate the presence of telomere. Yellow dumbbell shapes represent the locations and sizes of centromeric regions. **B** Characterization of the centromere on Chr12 of the hapB. The histogram shows Class I retrotransposons, Class II DNA transposons, gene and tandem repeat density of Chr12. The heatmap shows pairwise similarity of the 50 Kb sequence along the whole chromosome of Chr12

The telomere is a highly repetitive DNA region at the end of the chromosome, which protects chromosomes from fraying or tangling (Shakirov et al. 2022). In plants, the telomere sequences are highly conserved in unique repetitive 7-bp nucleotide units (CCCTAAA at the 5’ end and TTTAGGG at the 3’ end) (Fajkus et al. 2005). Using the TeloExplorer module of quarTeT (Lin et al. 2023), 38, 38, 33 and 33 distinct telomeres were detected in the individual haplotypes of LC2 v1.0, and there are 12, 13, 11 and 9 chromosomes with telomeres presented at both ends, respectively (Table 1, Fig. 2A and Table S6).

Centromeres are pivotal in maintaining chromosome integrity and the accuracy of chromosome segregation during cell proliferation. Although they also compose of tandem repeat sequences, the repeat monomers and chromosome locations manifest a wide range of variations (Malik and Henikoff 2009). Plant centromeres display a surprisingly large structural diversity, not only in the number of repeat monomers and locations on individual chromosomes but also in DNA sequences of repetitive monomers (Comai et al. 2017; Melters et al. 2013). Recent studies have shown that the repeat type of the centromere region in kiwifruit is complex, containing a major repeat monomer and several minor repeat monomers (Han et al. 2023; Wang et al. 2023; Yue et al. 2023). Based on an identification of the centromeric regions in the LC2 v1.0 by using the CentroMiner module of quarTeT (Lin et al. 2023), we found that the majority of the centromeres were located near to the middle parts of the chromosomes, while minors were resided at the chromosomes’ terminals, including Chr15, Chr26 and Chr27 (Fig. 2A and Table S7). In addition, we conducted comprehensive analyses to validate the region of the centromere and found that class I retrotransposons were primarily resided in traditionally heterochromatic regions such as centromeric, pericentromeric and subtelomeric regions, as well as that class II DNA transposons were evenly distributed across the genome and tandem repeats were predominately located in the centromere region (Fig. 2B), which are consistent with previous observations (Ma et al. 2007; Perumal et al. 2020). These results implicate the length of the majority centromere monomers are relatively conserved in *Actinidia* species (Wang et al. 2023; Yue et al. 2023). Nevertheless, our analyses were completely based on bioinformatics predictions without any experimental demonstrations, and their accurate positions need to be further validated by more robust techniques such as ChIP-seq (Chen et al. 2023).

### Allele-specific expression caused by structural variations between haplotypes

A comparative analysis between the individual haplotypes showed that they are characterized with a set of similar genomic features, such as close genome sizes, parallel repeat contents, and similar gene numbers (Table 1). Subsequent whole-genome alignments further revealed a highly conserved synteny present in the main body of the individual haplotypes (Fig. 2A). In comparison with hapA, 3,625,756, 3,512,652, 3,520,356 single-nucleotide polymorphisms (SNPs), 254,509, 248,027, 247,091 insertions, 255,262, 247,065, 246,677 deletions, 125, 120, 103 inversions, 618, 568, 624 translocations, and 213, 304, 248 duplications, respectively, were detected in hapB, hapC, and hapD. Specifically, variations in SNPs and small insertions or deletions (InDels) may result in either frameshifts or changes in start/stop codons, splice sites, or encoded amino acids (Fig. 2A and Table S8). In addition, 157,658 shared genes (40,859 from hapA, 40,063 from hapB, 38,638 from hapC and 38,098 from hapD) belonging to 35,563 orthologous gene families were obtained from four haplotypes, representing a core set of gene clusters in LC2 v1.0 (Table 1 and Table S9). By contrast, 1,404, 1,314, 1,195 and 1,124 genes were annotated specifically to the four individual haplotypes (Table 1).

Then we conducted allele-specific annotation. The homologous genes located at the same locus on homologous chromosomes are defined as alleles in autopolyploid genomes (Osborn et al. 2003). Using JCVI program (Tang et al. 2008), we totally annotated 146,870 genes with alleles defined in the whole genome, including 21,091 loci with four alleles, 7,951 with three, 9,722 with two. 35,034 genes (9,983, 9,162, 7,816 and 8,073 in four individual haplotypes, respectively) has no allele detected, which was defined as “singletons” in this study. We also investigated expression levels of genes in each homologous chromosome group, and detected no significant overall allelic dominance in the expression profiles of the *A. arguta* (Figure S2A).

Based on the assembled haplotypes and RNA-seq data, we investigated the gene sequence divergence and expression imbalance of allelic genes. We took a total of 21,091 loci with four allelic genes as an example (Table S10). Based on the analysis of 12 RNA-seq datasets, 1,142 out of 21,091 four-allele loci (5.4%) exhibited significantly allele-specific expression (ASE) pattern (*P*-value < 0.05 and FDR < 0.05) throughout different storage stages of the ‘Longcheng No.2’ fruits, showing that majority of four-allele genes were expressed in a balanced manner. Out of 1,142, 137 are the consistent allelic specific expression genes (ASEGs) with biased expression toward a single allele in all samples. By contrast, we found 1,005 inconsistent ASEGs that displayed a switched expression magnitude among alleles in different storage stages (Figure S2B and Table S11). This indicated that most ASEGs that displayed alternation of expression bias among alleles in different storage stages, consistent with that observed in rice (Shao et al. 2019). Gene ontology (GO) analysis showed that ASEGs are significantly enriched in multiple biological processes, such as metabolic process (GO:0008152), catalytic activity (GO:0003824) (Table S12). Kyoto Encyclopedia of Genes and Genomes (KEGG) pathway annotation revealed that the ASEGs were functionally enriched in multiple biological processes, including endocytosis (ko04144), plant-pathogen interaction (ko04626) and thermogenesis (ko04714) (Table S13), suggesting that a potential mechanism to overcome deleterious mutations occurred in important genes related to basic biological functions.

### Phylogenetic analysis reveals evolutionary history of the tetraploidization

**Fig. 3:**
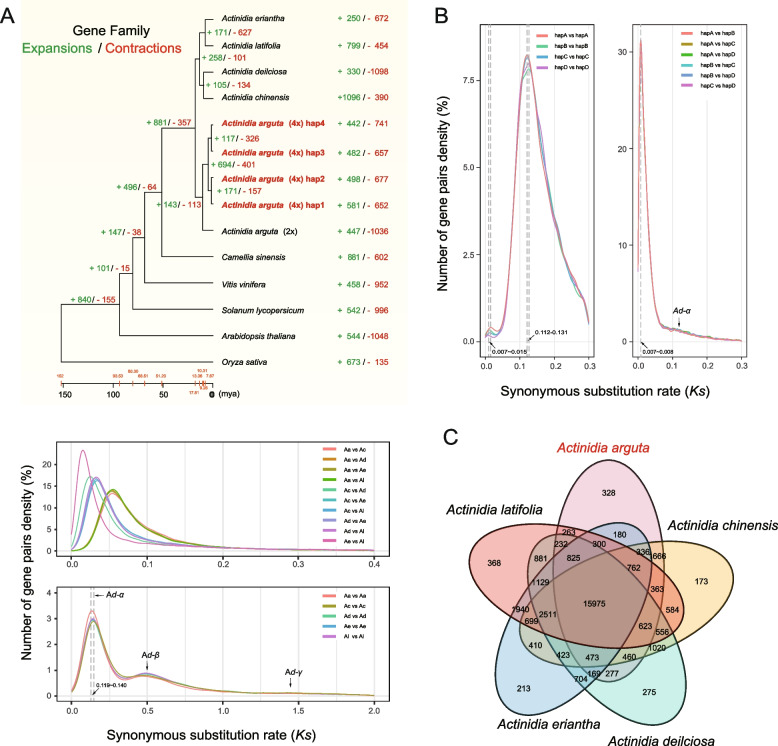
Phylogenetic relationships, comparative genomics, and evolutionary analyses of *Actinidia arguta*. **A** The phylogenetic tree showing gene family expansions/contractions and divergence time (top panel), *K*_*s*_ values of orthologous genes pairs (middle panel) or estimating for whole genome duplication (WGD) of *Actinidia* species including *A. arguta* (Aa), *A. chinensis* (Ac), *A. deliciosa* (Ad), *A. eriantha* (Ae) and *A. latifolia* (Al) using paralogous genes pairs (bottom panel). 2x or 4x represents diploid or tetraploid, respectively. The phylogenetic tree was constructed for eleven species based on the orthologous genes. Species divergence time was calculated by r8s, and the expansions or contractions of gene families were analyzed using CAFÉ 5. **B** *K*_*s*_ values calculated from paralogous pairs (left panel) and allelic pairs (right panel) throughout the four individual haplotypes. **C** Venn diagram of gene families from five *Actinidia* species

Using Orthofinder and r8s (Sanderson 2003), we inferred the phylogenetic position and divergence times among *A. arguta* and ten other plant species, including diploid (2x) (Akagi et al. 2023) and tetraploid (4x) *A. arguta* (Aa), *A. chinensis* (Ac) (Yue et al. 2023), *A. deliciosa* (Ad) (Xia et al. 2023), *A. eriantha* (Ae) (Wang et al. 2023) and *A. latifolia* (Al) (Han et al. 2023), *Camellia sinensis* (Zhang et al. 2021), *Solanum lycopersicum* (Zhou et al. 2022), *Vitis vinifera* (Jaillon et al. 2007), *Arabidopsis thaliana* (Lamesch et al. 2012) and *Oryza sativa* (Ouyang et al. 2007). The resultant phylogenetic tree showed that the *A. arguta* was diverged from other four *Actinidia* species at ~ 17.81 Mya, and distinct expansion or contraction in 694 or 401 gene families were detected (Fig. 3A top panel). Using JCVI (Tang et al. 2008), 276,984 orthologous pairs among the five *Actinidia* species were identified. The synonymous substitution rate (*K*_*s*_) analysis of orthologous gene pairs consistently demonstrated that *A. arguta* speciated earlier than the other species (Fig. 3A middle panel and Table S14), and the *K*_*s*_ of 79,331 paralogous pairs indicated possibly two recent WGD events occurred at 17.6 ~ 20.6 Mya (*Ad-α*) or ~ 73.7 Mya (*Ad-β*), respectively (Fig. 3A bottom panel and Table S15). These results are largely consistent with previous analyses for *A. chinensis* (Huang et al. 2013; Shi et al. 2010) and *A. deliciosa* (Xia et al. 2023).

Using JCVI software (Tang et al. 2008), 14,548, 14,592, 13,885 and 13,552 paralogous pairs in four individual haplotypes, respectively, were identified. Analyses of the *K*_*s*_ values of these paralogous pairs revealed that a large-scale duplication event occurred 1.03 ~ 2.21 Mya after *Ad-α* (Fig. 3B left panel and Table S16), the possible date of tetraploidization in *A. arguta* cv. ‘Longcheng No.2’. The *K*_*s*_ values estimated for the allelic genes (including 21,091 loci with four alleles, 7,951 with three and 9,722 with two) peaked at 0.007, consistently suggesting tetraploidization of *A. arguta* was happened at ~ 1.03 Mya (Fig. 3B right panel and Table S17).

In addition, 328 specific gene families containing a total of 1,152 genes were annotated in the *A. arguta* using Orthofinder (Emms and Kelly 2019), including a number of biotic/abiotic resistance genes, such as NBS-LRRs and RLK-LRRs gene families (Table S18). GO analysis showed that the specific genes are enriched in multiple biological processes and molecular functions, such as metabolic process (GO:0008152), catalytic activity (GO:0003824) and response to stimulus (GO:0050896) (Figure S3A and Table S19). Functional enrichments in multiple biological processes were identified, including environmental adaptation and signal transduction pathway in KEGG (Figure S3B and Table S20).

### Expansion of NBS-LRR and CBF gene families by tetraploidization enhances environmental adaptation

**Fig. 4:**
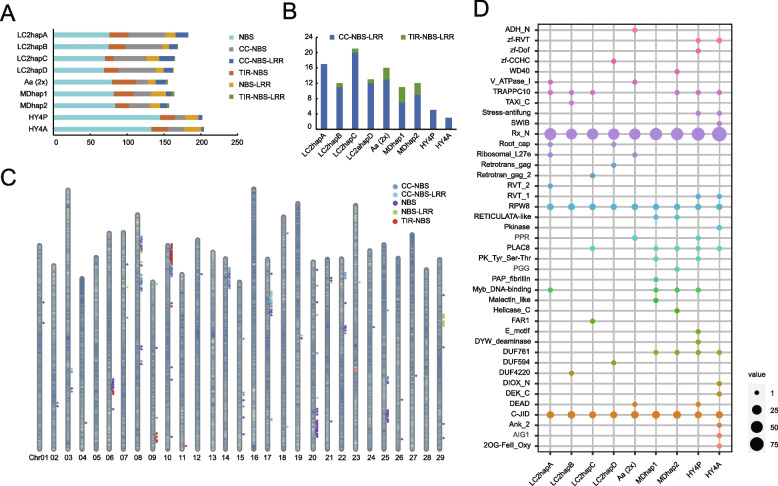
Genome-wide identification of NBS-LRR gene family in *Actinidia arguta*, *A. chinensis* and *A. eriantha*. **A** Classification of the NBS-LRR genes in the different genomes. Six colors represent different types of NBS-LRR genes. **B** The number of TNL or CNL type in different genomes. **C** Chromosomal distribution of NBS-LRR genes in hapA of *A. arguta* (4x). **D** The integrated domains of NBS-LRR genes in the different genomes

The nucleotide-binding site and leucine-rich repeat receptors (NBS-LRRs, NLRs) represent a family of highly diverse genes functioning in plant immunity by specifically recognizing pathogen effectors (Van de Weyer et al. 2019). We employed the assembled *Actinidia* genomes to analyze NLR diversity. There are 162, 146, 149, and 146 NLRs annotated in four individual haplotypes of *A. arguta* (4x), 143 in the monoploid genome derived from an *A. arguta* (2x) accession (Akagi et al. 2023), 161 and 153 in two individual haplotypes of *A. eriantha* (Wang et al. 2023), as well as 198 and 200 in two individual haplotypes of *A. chinensis* (Yue et al. 2023) (Fig. 4A). Based on the presence/absence of Toll/Interleukin-1 receptor (TIR), coiled-coil (CC) and LRR domains, these genes can be further classified into six groups, i.e., NBS, TIR-NBS, CC-NBS, NBS-LRR, CC-NBS-LRR (CNL) and TIR-NBS-LRR (TNL). The majority of CNL and TNL proteins were shown to serve as pathogen detectors, either by directly interacting with pathogen effectors or by monitoring changes in the condition of host proteins that are targeted by these effectors (Kourelis and van der Hoorn 2018). And the N-terminal TIR and coiled-coil (CC) domains were implicated in regulating the oligomerization and activation process of NLR proteins (Collier et al. 2011; Schreiber et al. 2016). Several studies showed that both dicotyledonous and monocotyledonous plant genomes encode CNL proteins, while TNL proteins are absent in monocotyledonous plants and several eudicots (Collier et al. 2011; Jacob et al. 2013; Shao et al. 2016). Interestingly, we found the total number of TNLs and CNLs in *A. chinensis* is significantly lower than that observed in *A. eriantha* and *A. arguta*, and importantly, the TNL genes were not detected in the tested genome of *A. chinensis* (Fig. 4A, B).

NLRs tend to cluster together in the genome and contribute to plant defense (Van de Weyer et al. 2019). We also observed that the NLR genes are predominantly distributed on the chromosomes in clusters (Fig. 4C). Specifically, five large NLR clusters are located on chromosomes 6, 8, 10, 14, 17 and 20. The prevalence of a particular type of NLRs within a single large NLRs cluster, suggesting that the clustered NLR genes may have originated from a common ancestor by tandem duplications (Fig. 4C). In addition, we identified the integrated domain architectures which may be related to proteins that were repeatedly affected by pathogens (Wessling et al. 2014). And our analysis showed that Rx_N, TRAPPC10, RPW8, PLAC8 and C-JID are universally present in all tested *Actinidia* species, and that several domains are specific to *A. arguta* (4x), such as Zf-CCHC, V_ATPase_I, Taxi_c, FAR1 (Fig. 4D).

**Fig. 5:**
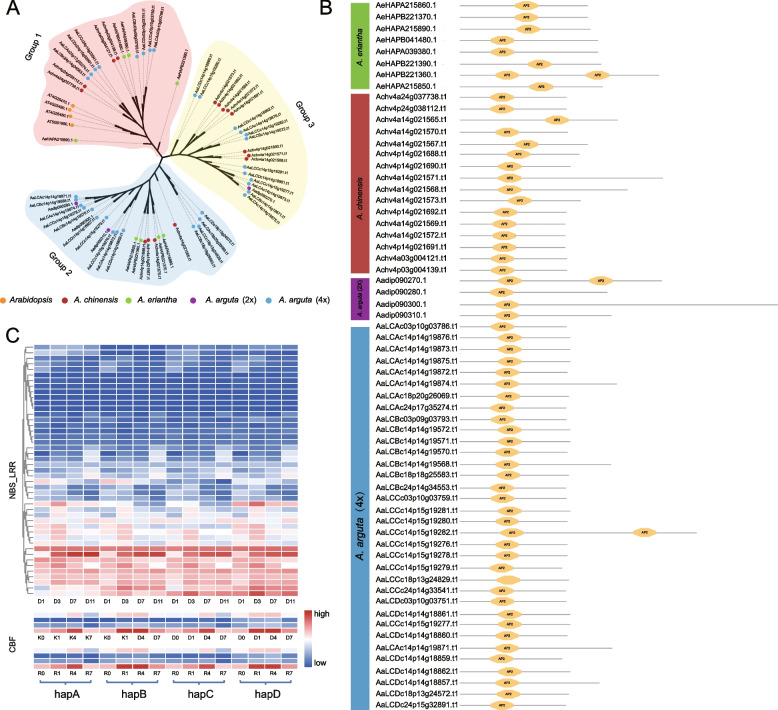
Genome-wide identification of CBF gene family in *A. arguta* and four-allele genes expression pattern in different gene families. **A** Phylogenetic tree of CBF genes in *A. arguta*, *A. chinensis*, *A. eriantha* and *Arabidopsis thaliana*. 2x or 4x represents diploid or tetraploid, respectively. **B** Protein domain analysis of CBF genes. **C** The expression patterns of four-allele genes in NBS-LRR at different storage stages (1, 3, 7, 11 days post-harvest), and CBF gene families in different cultivars ‘Kuilv male’ (K) and ‘Ruby-3’ (R) under different durations (0, 1, 4, 7 h of –25°C) of frozen-treated. **D** represent day(s) after postharvest

Members of transcription factor (TF) C-repeat binding factor (CBF)/dehydration-responsive element binding (DREB1) subfamily has been known to play a critical role for plants to respond to various abiotic stresses, such as freezing, drought, and salinity (Thomashow 1999). Using a genome-wide identification we found that *A. chinensis* (2x), *A. eriantha* (2x), *A. arguta* (4x) or *A. arguta* (1x, a monoploid) encode 16, 8, 34, or 4 CBF genes, respectively (Fig. 5A). All CBF genes annotated contain the AP2 domain and other conserved flanking sequences on both sides (Fig. 5B). Phylogenetic analysis revealed that these CBF genes can be classified into three groups, and most CBF genes derived from *A. arguta* (2x or 4x) belong to Group 3 (Fig. 5A). Group 1 consists only of CBF genes from *A. eriantha* and *A. chinensis.* We used multiple transcriptome data of *A. arguta* to investigate the expression differences between the alleles of NBS-LRR and CBF gene families, and we found that there were no significant differences in the expression patterns of alleles of these gene families (Fig. 5C). This suggests that subgenomic dominance does not exist in tested *A. arguta* tetraploid.

### Construction of texture-related regulatory network by WGCNA

**Fig. 6:**
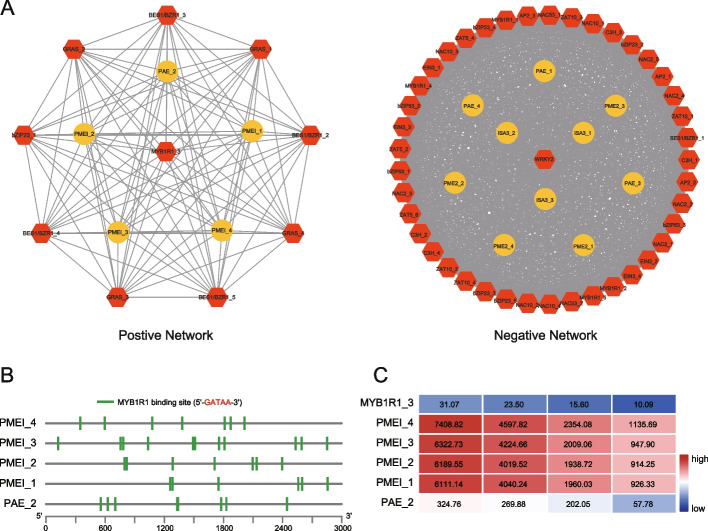
The regulatory network involved in mediating fruit texture of *Actinidia arguta*. **A** Diagram visualizing the positive or negative network regulating fruit texture. Yellow circles or red hexagons represent structural genes or transcription factors, respectively. **B** Analysis of the promoters of the structural genes (Pectin methylesterase inhibitors: PMEI, Pectin acetylesterase: PAE) in positive network. **C** The expression pattern of candidate genes and transcription factors screened by WGCNA. Red or blue represents high or low expression, respectively

As a fruit with typical respiratory climacteric, *A. arguta* is prone to softening and rotting due to its thin and easily broken skin. Therefore, we measured several postharvest quality indicators of kiwifruit stored at ambient temperature of 25 °C every two days, including ethylene release rate, texture, Brix, acidity and water loss. Transcriptomic datasets were generated using four different fruit samples of ‘Longcheng No.2’ (DPH; days post-harvest 1, 3, 7 and 11) were sampled to explore the potential regulatory network during postharvest softening (Table S21). Weighted correlation network analysis (WGCNA) was conducted by integrating the transcriptome datasets (Fig. 6A and Figure S4).

Module-trait relationships (MTRs) differ in terms of physiological changes during different storage periods. These modules contain positively and negatively related genes whose expression levels fluctuate between periods. Modules with MTR > 0.85 were selected as representatives for further analysis and two modules were selected for the fruit texture. The results revealed that both MEturquoise (*r* = -0.97, *p* = 1.4e^−7^) and MEblue (*r* = 0.87, *p* = 2.2e^−4^) were highly correlated with the fruit texture alterations (Figure S4B). The WGCNA-defined hub genes from MEturquoise and MEblue were selected for further analysis (Figure S4 and Table S22).

Based on previous studies and the present genome annotations, we selected the candidate hub genes involved in the regulation of fruit texture (Shi et al. 2022), including structural genes, such as pectin acetylesterase (PAE), pectin methylesterase inhibitors (PMEI), isoamylase3 (ISA3) and *β*-galactosidases (TBG), as well as transcription factors, such as WRKY, BES1, NAC, MYB, MYC, TCP, AP2/ERF, ZF, HLH, GRAS (Fig. 6A). The regulatory network showed that the transcription factors MYB1R1_3 (AaLCBc26p08g36658) were associated with the structural gene PMEI_1 (AaLCAc24p17g35206), PMEI_2 (AaLCBc24p14g34497), PMEI_3 (AaLCCc24p14g33485), PMEI_4 (AaLCDc24p14g32827) and PAE2 (AaLCBc09p16g13019) in a positive regulatory module for the fruit texture (MEblue), and *cis*-acting element analysis showed that a number of MYB1R1 binding sites (5'-GATAA-3') in promoter region (3 kb upstream) of the structural gene were identified (Fig. 6B and Table S23). These observations were further verified through correlating changes of expression patterns between the transcription factors and their putative direct targets (Fig. 6C).

## Discussion

It is a huge challenge to assemble a multiple ploidy genome, particularly the homologous polyploids, due to the large amount of sequencing data required and the difficulty in chromosome phasing, especially distinguishing highly similar sequences between homologous chromosomes. To address the assembly issue of highly repetitive and highly homologous genomic regions of *A. arguta* tetraploid, we employed deep sequencing platforms to obtain highly accurate PacBio HiFi long reads and chromosome conformation capture sequencing (Hi-C) data, and consequently achieved a haplotype-resolved genome containing four haplotypes (Figs. 1 and 2). The unitig N50 is 0.94 Mb that has been substantially improved as compared to other assembled tetraploid genomes such as sugarcane (Zhang et al. 2018) and cultivated alfalfa (Chen et al. 2020). It is worth mentioning that we employed the genome sequence derived from a diploid *A. arguta* (Akagi et al. 2023) as a reference to anchor and determine the position and order of the unitigs onto the syntenic regions against the four sets of the homologous chromosomes in our *A. arguta* tetraploid, and the resultant assembles were further validated by Hi-C heatmaps. The chromosome orientation was also adjusted by comparison with the high-quality T2T genome of HY4P derived from the diploid *A. chinensis* cv. ‘Hongyang’ (Yue et al. 2023). Consequently, the generated genome of *A. arguta* cv. ‘Longcheng No.2’ consists of four haplotypes with 682-764 gaps and an LAI ranging from 16.86 to 17.62, indicating the high continuity and integrity of the genome assembled.

Whole-genome duplication (WGD) followed by gene loss and neofunctionalization has been regarded as an important driving force for plant species diversification, contributing to genetic innovation and adaptation to extreme environments (Landis et al. 2018; Soltis et al. 2015). Majority of eudicots including kiwifruit share at least one ancient WGD event (γ) and two recent lineage-specific WGDs (*Ad-α* and *Ad-β*) (Akoz and Nordborg 2019; Blanc et al. 2000; Huang et al. 2013; Vision et al. 2000; Xia et al. 2023). In the present study, three WGD events (Fig. 3A bottom panel) predating the tetraploidization of the *A. arguta* were analyzed and the time estimates are roughly similar to that calculated in *A. chinensis* (Huang et al. 2013) and *A. deliciosa* (Xia et al. 2023). Phylogenetic analysis (Fig. 3A the top panel) and synonymous substitution rate (*K*_*s*_) calculation using orthologous genes pairs between the sequenced *Actinidia* species (Fig. 3A middle panel) consistently suggest that the divergence time of *A. arguta* was earlier than others. All the *K*_*s*_ values analyzed for paralogous genes pairs within the individual haplotypes are peaked at 0.007 ~ 0.015 (Fig. 3B left panel), implicating that a more recent WGD event (i.e., tetraploidization) possibly occurred in the *A. arguta* after *Ad-α* event (17.6 ~ 20.6 Mya)*.* From this analysis we inferred that the newly detected WGD event might result from the tetraploidization occurred 1.03 ~ 2.21 Mya. Consistently, calculation of *K*_*s*_ across allelic genes pairs also demonstrated the spontaneous tetraploidization of the *A. arguta* is estimated to have formed ~ 1.03 Mya following the most recent WGD event (A*d-α*) occurred 18.7 Mya (Fig. 3B left panel). Although a *K*_*s*_ peak reasonably provides evidence for the existence of a polyploidization event, but it does not truly give the age of this event, because the distance between homoeologues pairs combines the divergence since polyploidy with the pre-existing divergence between diploid progenitor homoeologues. Therefore, peak represents the mode of *K*_*s*_ values of all the homoeologues from both the diploid progenitor and the resultant polyploid, and the date calculated for the *K*_*s*_ peak is an over-estimated age of polyploidization. By contrast, a large number of genes pairs originally generated from local duplications (like tandem) after tetraploidization might attenuate the age of the estimated WGD event (Han et al. 2022). Finally, it worth noting that this investigation includes only single-genome samples from *A. arguta* tetraploid cv. ‘Longcheng No.2’ and that these samples are not likely to represent plants from the actual ancestor populations that may independently give rise to the polyploidization. Therefore, further analyses of broad samples including additional distinct *A. arguta* tetraploids with wide range of geographic distributions are needed to improve the accuracy of the polyploidization time estimates.

There might be a debate regarding whether the *A. arguta* tetraploid is an autopolyploid or an allopolyploid. It has been demonstrated that, in allopolyploid, one subgenome with less sequence loss often predominates over the other(s) by displaying higher homologous gene expression (Murat et al. 2017; Yu et al. 2021). Nevertheless, our analyses indicate that the four sets of assembled haplotypes for *A. arguta* cv. ‘Longcheng No.2’ are highly similar in terms of size, gene count, and repetitive sequences content (Table 1). Six graphs based on *K*_*s*_ analysis of allelic genes pairs across 182,088 loci are overlapped (Fig. 3B right panel), suggesting a high homogeneity between the individual haplotypes assembled. And there is no significant allelic dominance in expression profiles of tetraploid *A. arguta* (Figure S2A). All these results collectively suggest that *A. arguta* cv. ‘Longcheng No.2’ used in this study is a stable, random pairings autotetraploid species, not returning to diploid state accompanied with massive gene loss after whole genome duplication (Chen et al. 2020; Julier et al. 2003).

It is interesting to inspect the chromosome structure variation after tetraploidization of *A. arguta*. Our analysis focused on totally 35,034 singletons across the four haplotypes that have no allelic genes. A total of 5834 (17%) of them were found highly homologous (> 80% similarity) to genes that have four alleles, in which 2726 (47%) genes remain on the same homologous chromosomes group, and 3108 (53%) genes moved to non-homologous chromosomes. A large proportion of these genes might be originally generated from a local duplication (like tandem) after tetraploidization. 3963 (11%) or 5146 (15%) singletons were identified homologous to genes that have two or three alleles, in which 2030 (51%) or 2888 (56%) were resided in the same homologous chromosomes group while 1933 (49%) or 2258 (44%) moved to non-homologous chromosomes, respectively. We speculated that this type of singletons may be resulted from tetraploidization and subsequent inversion or translocation or both. A total of 15,574 (44%) singletons appeared to be highly homologous paralogs, and out of them 11,401 (73%) are located on the homologous chromosomes. These genes may be once allelic or originated from interchromosomal duplications after tetraploidization. An overall 7 ~ 16% inversion rate was observed in the genes described above. Finally, no highly homologous genes (> 80% similarity) were found for 4517 (13%) singletons, and 1976 of them are functionally annotated. Comparably, Hongyang v4.0 possesses 16,016 singletons, of which 7096 (44%) seems to be homologous paralogs and 4270 (60%) of them are located on the homologous chromosomes with a 5% inversion rate (Yue et al. 2023), indicating significant difference in the rate of structural variation between the diploid and tetraploid. These analyses suggest that intensive rearrangements and genetic variations occurred following the presence of tetraploid genome of *A. arguta.*

Importantly, polyploidization has been assumed to provide important genetic reservoir for successful plant domestication, implicating the importance of such events in agricultural practice (Moharana and Venancio 2020; Salman-Minkov et al. 2016). Our study found that there were 328 specific gene families (Fig. 3C) annotated in the tetraploid *A. arguta*, including NBS-LRRs (NLRs) and RLK-LRRs involving immune responses or stress resistance. In the case of *A. arguta* (4x), polyploidization directly leads to a doubling of the number of disease resistance genes (NBS-LRRs) (Fig. 4) and consequently may confer a reinforced capacity against pathogens. *Pseudomonas syringae* pv. *actinidiae* (Psa) is the causal agent of bacterial canker disease of kiwifruit that spreads rapidly throughout the world’s cultivated area, particularly to those growing cultivars of *A. chinensis* (Hemara et al. 2022). By contrast, *A. arguta* was reported to display strong resistance conferred by recognition of effectors delivered by Psa (Yoon and Rikkerink 2020). Our work suggests the possible reason making this difference is that *A. chinensis* encodes NLRs either truncated or lacking functional domains (Fig. 4). Phylogenetic analysis of the CBF gene family revealed that group3 is unique to *A. arguta* compared with *A. chinensis* and *A. eriantha*. These genes are likely to be the key genes that make *A. arguta* more cold-tolerant compared to the other *Actinidia* species (Fig. 5).

The rapid softening of kiwi berry after harvest, leading to poor storage ability, is a major limiting factor for its widespread commercialization. The softening of the fruit is primarily caused by the degradation of cell wall components as well as the conversion of starch into soluble sugars during the ripening process (Shi et al. 2022). Previous studies have indicated that structural genes, such as pectinesterase (PE), polygalacturonase (PG), *β*-galactosidase (TBG), xyloglucan endotransglycosylase (XET), and amylase, play pivotal roles in the softening process during fruit ripening (Bonghi et al. 1996; Wegrzyn and MacRae 1992). Several transcription factors involved in the regulation of fruit softening have also been identified, such as *AdDof3* (Zhang et al. 2018), *AdZAT5* (Zhang et al. 2022), *AdEIL2* and *AdEIL3* (Yin et al. 2010). Our WGCNA analysis revealed a positive correlation between the expression of both *PMEI* and *PAE* genes and the fruit texture indicators during ripening process of *A. arguta*. Further analysis reveals that transcription factors MYB1R1 display a coordinate expression patterns with the structural genes, suggesting a potential regulatory relationship between them (Fig. 6).

In conclusion, we present a high-quality genome of tetraploid *A. arguta* for the first time, providing valuable resource for the kiwifruit biology studies and molecular breeding research.

## Materials and methods

### Plant materials, library preparation and DNA sequencing

Green wood cuttings of *A. arguta* cv. ‘Longcheng No.2’ were picked and grown in a tissue culture incubator at Anhui Agricultural University, Anhui Province, China, under 25 °C, 12-/12-h days. Fresh young healthy leaves were collected from 3-week-old branches, quickly frozen with liquid nitrogen and then stored at –80 °C for PacBio HiFi and Hi-C sequencing. High molecular weight genomic DNA (gDNA) was extracted separately from each leaf tissue sample using a slightly modified cetyltrimethylammonium bromide (CTAB) method (Allen et al. 2006). The quality and quantity of the isolated gDNAs were evaluated with an Agilent 2100 Bioanalyzer (Agilent Technologies, CA, USA) and a Qubit fluorometer instrument (Thermo Fisher Scientific, MA, USA), respectively. For PacBio HiFi sequencing, a standard SMRTbell library was prepared with 50 μg gDNA using the SMRTbell Express Template Prep Kit 2.0 according to the manufacturer's instructions. SMRTbell libraries were then sequenced on the PacBio Sequel II system (Pacific Biosciences, CA, USA). The Hi-C sequencing library was prepared and sequenced based on a previously published protocol (Rao et al. 2014).

#### Determination of quality-related physiological indexes and polyploid identification

Fruits of ‘Longcheng No.2’ were harvested at 140 DAP (days after pollination) and then stored in a cabinet at 25°C. Acidity, brix, texture, ethylene release rate, and water loss were measured every two days. Fruits were placed in a 2.3 L airtight container, sealed for 2 h and then the ethylene production rate was measured using the gas chromatograph (Agilent 7890B GC System, USA). The chromatographic column was Agilent 19095P-QO4: 3 HP-PLOT Q, 30 m × 530 μm × 40 μm. The FID detector parameters were set as follows: heater at 150 °C, airflow at 400 ml·min^−1^, hydrogen flow at 30 ml·min^−1^, and tail gas flow at 15 ml·min^−1^. The initial temperature of the column chamber was set at 40°C and the maximum temperature was 290°C. A standard curve was constructed using ethylene standard gas. Then 5 mL of gas from the container was drawn and injected into the gas chromatograph. Each sample was measured three times, and the average was taken as the result. The Brookfield™ CT3 texture analyzer (Brookfield Middleboro, USA) with an TA39 adaptor (2 mm knife edge) was used to determine the hardness of fruit flesh. The probe was driven to a depth of 5 mm at a speed of 0.5 mm·s^−1^. Hardness was measured at four opposite positions per flesh, and the maximum force measured was expressed in Newton (N). Brix and acidity were measured with a hand-held refractometer (PAL-BX, ACID-8, ATAGO, Japan). Once finished, the fruit were frozen immediately in liquid nitrogen, and then stored at − 80°C until used. Ploidy identification was performed using young leaves collected from regenerating shoots according to the method described in a previous study (Jin-Hu et al. 2002). The experiments were performed using a BD FACSCalibur flow cytometer (BD Biosciences, USA) and the results were analyzed using the BD FACSCalibur software package.

#### Genome assembly and assessment

The raw data generated by the PacBio Sequel II system were processed using the SMRT Analysis software suite (version 5.1.0; https://www.pacb.com/products-and-services/ analytical-software/smart-analysis/). The consensus HiFi reads were generated by the CCS subprogram (https://github.com/PacificBiosciences/ccs) with default parameters. Subsequently, the CCS reads were de novo assembled into unitigs to get a draft assemble using the hifiasm (v0.18.5) software (Cheng et al. 2022). The allelic unitigs were identified using the diploid *Actinidia arguta* genome (Akagi et al. 2023) as a reference. Then the Hi-C reads were mapped to the draft assembly using the bwa aln algorithm. Based on Hi-C signals, the partition function of the ALLHiC software was used to categorize all unitigs into 29 clusters. For each clusters, the weaker signals and signals that link alleles were trimmed using the prune function within the ALLHiC software, based on Hi-C signals and allelic unitig information. Then unitigs in each cluster were assign to four/eight groups using the function of partition with ALLHiC. Unplaced unitigs were then assign into partitioned groups using the rescue function in ALLHiC. After that, all the unitigs in each groups were ordered and orientation using the optimize with ALLHiC.

The Hi-C contact signal within and between groups were manually checked and adjusted using Juicebox (Durand et al. 2016) software. Finally, a total of 116 pseudochromosomes were generated and successfully divided into four haplotypes.

Multiple strategies were used to evaluate the assembly quality of the genome. The phasing quality of haplotypes were validated by the KAT program (Mapleson et al. 2017) with default parameters. The completeness of the genome assembly was estimated using the BUSCO program (version 5.2.1) (Manni et al. 2021) with the Embryophyta OrthoDB v10 dataset (https://www.orthodb.org). Genome assembly continuity was evaluated based on unitig N50 values and LAI (Ou et al. 2018). Additionally, the HiFi and Hi-C reads were realigned to the assembly to calculated the mapping ratio and evaluate the reliability of genome assembly.

#### Repeat identification and gene annotations

The transposable elements (TEs) were annotated using the comprehensive pipeline EDTA (Ou et al. 2019) with default parameters. The tandem repeats (TRs) were identified by TRF software (2 7 7 80 10 50 500 -f -d -m) (Benson 1999). All the four assemblies were soft-masked by RepeatMasker (Tarailo-Graovac and Chen 2009).

Gene structural annotation was performed by the BRAKER v3.0.0 (Gabriel et al. 2023), which combines the evidence of de novo prediction, transcript and homology protein. A total of 12 RNA-seq datasets (Table S24) were provided to assist gene prediction in the present study. For gene function annotation, the eggNOG-mapper was used to against a series of protein sequence databases (Cantalapiedra et al. 2021). GO and KEGG enrichment analyses were performed using the R package clusterProfiler (Yu et al. 2012). The OrthoFinder program (version 2.5.4) (Emms and Kelly 2015) was used for the identification of gene families between the primary haplotype and three alternate haplotypes.

#### Telomere detection and centromere localization

The quarTeT toolkit (Lin et al. 2023) was used to detect the location of telomere and centromere. The normalized and unified sequence AAACCCT within 50 kb of each terminal chromosome sequence was identified as a telomere. The continuous tandem repeat-rich areas were assumed to be centromere candidates. Meanwhile, the centromeric monomer was defined as the monomer that occupied the majority of each centromere. The length distribution of kiwifruit centromeric monomers was measured after getting all of the centromeric monomers, and the most frequent monomer was defined as the representative monomer in this study.

#### Genome comparison and synteny analysis

Pairwise comparisons of the four halotype assemblies were conducted using the MUMmer toolbox (version 4.0.0beta2) (Marçais et al. 2018) with the following parameters: -maxmatch -c 500 -b 200 -l 100. Subsequently, the alignment results were filtered using the delta-filter with parameters (-m -i 90 -l 100) and show-snps util was used to obtain the SNP and InDel information. Finally, mummerplot was used to generate a dot plot representing each comparison. Meanwhile, we introduced Synteny and Rearrangement Identifier (SyRI) (Goel et al. 2019) to identify collinear orthologs, structural variations, and sequence differences baed on the alignment results from MUMmer.

#### Transcriptome sequencing and analysis

Samples from 1 DPH (days post-harvest), 3 DPH, 7 DPH, and 11 DPH were selected for RNA-sequencing. Three biological replicates were collected for each sample. Total RNA was extracted from the samples, and messenger RNA (mRNA) libraries were constructed and sequenced using the Illumina Novaseq 6000 platform. The clean reads were aligned to the genome of LC2 v1.0 using Hisat2 v2.1.0 (Guo et al. 2022). The gene expression levels were represented using transcripts per million (TPM) values and estimated using featureCounts (Liao et al. 2014). The differentially expressed genes (DEGs) were identified using DESeq2 (Love et al. 2014).

#### Determination of allele-specific expression

First, genome-wide alignment blocks between the four haplotypes were extracted from the synteny analysis. Second, the most similar gene pairs with the highest sequence similarity of coding proteins were identified using JCVI (Tang et al. 2008) and Then, *K*_*s*_ values between alleles are calculated using ParaAT (Zhang et al. 2012) and KaKs_Calculator 3.0 (Zhang 2022). A total of 12 RNA-seq datasets (Table S24) were aligned to the four haplotype genomes and then used to calculate the gene expression values using the method described earlier. ASEGs were determined using the criterion that the log_2_ (fold change) values of TPM between two alleles were greater than 1 and the *P* value < 0.05.

#### Gene co-expression network construction

Differentially expressed genes (DEGs) of the different storage periods (D1, D3, D7, D11) were selected to construct gene co-expression networks using the WGCNA package in R (Langfelder and Horvath 2008). The co-expression modules were identified using the automatic network construction function (blockwiseModules) with the following parameters: power set to 16, minimum module size of 50, module cuttree height of 0.25, and a maximum block size of 10,000. Module eigengenes were used to describe the most common gene expression models in each module. The KME value is based on the Pearson correlation coefficient between the expression level and module eigengenes. Then we selected the gene significance (GS) ≥ 0.8 and module eigengene-based connectivity (KME) ≥ 0.8 for the analysis of hubgenes in the gene co-expression network. The network was constructed using the Cytoscape software (Greenfest-Allen et al. 2017). The *cis*-acting elements in the promoter region of the structural gene were searched by PLACE (https://www.dna.affrc.go.jp/PLACE/) (Higo et al. 1999).

#### Construction of phylogenetic tree and gene family analysis

OrthoFinder (v2.5.4) (Emms and Kelly 2019) was used to identify orthologous groups between the ten representative species (*A. arguta* var. melanandra (Aa) (Akagi et al. 2023), *A. chinensis* Hongyang v4.0 (Ac) (Yue et al. 2023), *A. deliciosa* Acd (Ad) (Xia et al. 2023), *A. eriantha* MD (Ae) (Wang et al. 2023) and *A. latifolia* KY (Al) (Han et al. 2023), *Camellia sinensis* TGY (Zhang et al. 2021), *Solanum lycopersicum* ITAG5.0 (Zhou et al. 2022), *Vitis vinifera* v2.1 (Jaillon et al. 2007), *Arabidopsis thaliana* TAIR10 (Lamesch et al. 2012) and *Oryza sativa* v7.0 (Ouyang et al. 2007)). The soft r8s (v1.81) (Sanderson 2003) was used to estimate the species divergence time based on the information obtained from from TimeTree (http://www.timetree.org) (Hedges et al. 2015; Sanderson 2003). The analysis of gene family expansion and contraction was performed using the CAFÉ5 (v1.1, K = 4) program (Mendes et al. 2021), with the divergence times tree used as the input (Sanderson 2003). First, genome-wide alignment blocks between the four haplotypes were extracted from the synteny analysis. Second, the most similar gene pairs with the highest sequence similarity of coding proteins were identified using JCVI (Tang et al. 2008). Third, Ks values were calculated using ParaAT (Zhang et al. 2012) and KaKs_Calculator 3.0 (Zhang 2022). Subsequently, the Ks values were converted to divergence time according to the formula T = Ks / (2r), where T is the divergence time and r is the neutral substitution rate (*r* = 3.39 × 10^–9^). The specific genes of *A. arguta* were identified by Orthofinder (v2.5.4) (Emms and Kelly 2019) by comparing the whole protein sequence of four in *Actinidia* species (*A. chinensis* (Ac), *A. deliciosa* (Ad), *A. eriantha* (Ae) and *A. latifolia* (Al)).

#### Identification and analysis of the NBS-LRR, CBF gene family of *A*. *arguta*

The conservative domain annotation of all protein sequences was performed using PfamScan (https://www.ebi.ac.uk/Tools/pfa/pfamscan/). Those proteins including the NB-ARC domain were identified as candidate members of NBS-LRR. The coiled-coil domain of NBS-LRR proteins was predicted using Paircoil2 software (McDonnell et al. 2006). Based on the absence/presence of domains of NB-ARC, LRR, TIR, and CC, these members are divided into six groups including NBS, TIR-NBS, CC-NBS, NBS-LRR, CC-NBS-LRR (CNL) and TIR-NBS-LRR (TNL). Those proteins containing the AP2 domain and having conserved sequences of DSXWR and PKKPAGRKKFRETRHP on both sides were classified as candidate members of the CBF family.

### Supplementary Information

**Additional file 1:** **Figure S1.** Ploidy and phasing validation of *Actinidia arguta* cv. ‘Longcheng No.2’. (A) Ploidy analysis of *A. chinensis* diploid cv. ‘Hongyang’ (upper panel) and *A. arguta* tetraploid cv. ‘Longcheng No.2’ (lower panel) using flow cytometry. (B) Comparison of the amount of distinct K-mers absent and copy number variation between four haplotypes of LC2 v1.0 assembly and raw HiFi reads, respectively. **Figure S2. **Expression patterns of loci with four allelic genes. (A) Expression levels of four-allele genes among homologous chromosomes. The expression level was presented in transcripts per kilobase per million mapped reads (TPM). (B) The expression patterns of inconsistent (upper panel) and consistent (lower panel) allelic specific expression genes (ASEGs) at different storage stages (1, 3, 7, 11 days post-harvest) in four haplotypes. D represent day(s) after postharvest. **Figure S3. **GO and KEGG pathways enrichment analysis of 328 specific gene families in *Actinidia arguta *cv.‘Longcheng No.2’. (A) GO functional classification of specific genes. (B) KEGG pathway classification of specific genes. **Figure S4.** Identification of co-expression network modules in *Actinidia arguta *cv. ‘Longcheng No.2’. (A) Cluster dendrogram of genes subjected to any co-expression module. (B) Module-trait associations based on Pearson correlations. Red or blue color indicates a positive or negative correlation between the cluster and the trait, respectively.

**Additional file 2:** **Table S1. **Summary of the data sequenced by multiple sequencing technologies. **Table S2. **Statistics of scaffolds after the first round of tuning. **Table S3.** The unclosed gaps in pseudochromosomes in the four haplotypes. **Table S4.** The mapping rate of HiFi, Hi-C and RNAseq reads against the whole LC2 v1.0 genome. **Table S5.** Summary of annotated transposable elements in the four haplotype-resolved assemblies. **Table S6.** The identified telomeres in LC2 v1.0 of four haplotypes. **Table S7.** The identified centromeres in LC2 v1.0 of four haplotypes. **Table S8.** Annotation of genome structure variations between the primary hapA and other three haplotypes. **Table S9.** The shared genes between four haplotypes. **Table S10.** Protein-coding sequence identity of 21,091 loci with four, 7,951 with three and 9,722 with two alleles, as well as 35,034 singletons. **Table S11.** Protein-coding sequence identity of 1,142 ASEGs within 21,091 loci with four allelic genes. **Table S12.** The enriched GO terms for the ASEGs in the whole genome of LC2 v1.0. **Table S13.** The enriched KEGG pathway for the ASEGs in the whole genome of LC2 v1.0. **Table S14.** The orthologous gene pairs among *A. arguta*, *A. chinensis*, *A. deliciosa*, *A. eriantha* or *A. latifolia*. **Table S15.** The paralogous gene pairs between *A. arguta*(4x), *A. chinensis*, *A. deliciosa*, *A. eriantha* and *A. latifolia*. 4x represents tetraploid. **Table S16.** The paralogous genes in the four haplotypes of *A. arguta* (4x). 4x represents tetraploid. **Table S17.** The allelic genes in the four haplotypes of *A. arguta* (4x). 4x represents tetraploid. **Table S18.** The gene annotations for the specific gene families in the tetraploid *A. arguta*.**Table S19.** The enriched GO terms for the specific genes in the whole genome of the tetraploid *A. arguta*. **Table S20.** The enriched KEGG terms for the specific genes in the whole genome of the tetraploid *A. arguta*. **Table S21.** The indicators related to postharvest quality of fruit. **Table S22.** Texture-related turquoise or blue module of hubgenes. **Table S23.** The *cis*-acting elements in the hubgenes promoters. **Table S24.** RNA-seq datasets used in the analyses.

## Data Availability

All data generated or analyzed during this study are included in this published article.

## Abbreviations

*A*. *arguta*: *Actinidia* *arguta*
*A*. *chinensis*: *Actinidia* *chinensis*
*A*. *deliciosa*: *Actinidia* *deliciosa*
*A*. *eriantha*: *Actinidia* *eriantha*
*A*. *latifolia*: *Actinidia* *latifolia*
ASE: Allele-specific expression
CBF: C-repeat binding factors
DPH: Days post-harvest
DEG: Differentially expressed gene
DREB1: Dehydration-responsive element binding
GO: Gene ontology
InDel: Insertions or deletions
ISA3: Isoamylase
KEGG: Kyoto Encyclopedia of Genes and Genome
*Ks*: The number of substitutions per synonymous site
LAI: LTR assembly index
LTR: Long Terminal Repeat
NLR: Nucleotide-binding site and leucine-rich repeat receptors
PAE: Pectin acetylesterase
PMEI: Pectin methylesterase inhibitors
SNP: Single-nucleotide polymorphisms
TBG: *β*-Galactosidases
TEs: Transposable elements
TRs: Tandem repeats
WGCNA: Weighted gene co-expression network analysis
WGD: Whole genome duplications
ZF: Zinc finger
