# Supplementary material for: Haplotype-resolved genome assembly provides insights into evolutionary history of the Actinidia arguta tetraploid
Source: Mol Hortic. 2024 Feb 6;4:4. doi: 10.1186/s43897-024-00083-6 (PMC10845759; doi:10.1186/s43897-024-00083-6)
Supplement: Supplementary file 1 — Additional file 1: Figure S1. Ploidy and phasing validation of Actinidia arguta cv. ‘Longcheng No.2’. (A) Ploidy analysis of A. chinensis diploid cv. ‘Hongyang’ (upper panel) and A. arguta tetraploid cv. ‘Longcheng No.2’ (lower panel) using flow cytometry. (B) Comparison of the amount of distinct K-mers absent and copy number variation between four haplotypes of LC2 v1.0 assembly and raw HiFi reads, respectively. Figure S2. Expression patterns of loci with four allelic genes. (A) Expression levels of four-allele genes among homologous chromosomes. The expression level was presented in transcripts per kilobase per million mapped reads (TPM). (B) The expression patterns of inconsistent (upper panel) and consistent (lower panel) allelic specific expression genes (ASEGs) at different storage stages (1, 3, 7, 11 days post-harvest) in four haplotypes. D represent day(s) after postharvest. Figure S3. GO and KEGG pathways enrichment analysis of 328 specific gene families in Actinidia arguta cv.‘Longcheng No.2’. (A) GO functional classification of specific genes. (B) KEGG pathway classification of specific genes. Figure S4. Identification of co-expression network modules in Actinidia arguta cv. ‘Longcheng No.2’. (A) Cluster dendrogram of genes subjected to any co-expression module. (B) Module-trait associations based on Pearson correlations. Red or blue color indicates a positive or negative correlation between the cluster and the trait, respectively. [file 43897_2024_83_MOESM1_ESM.docx]

**
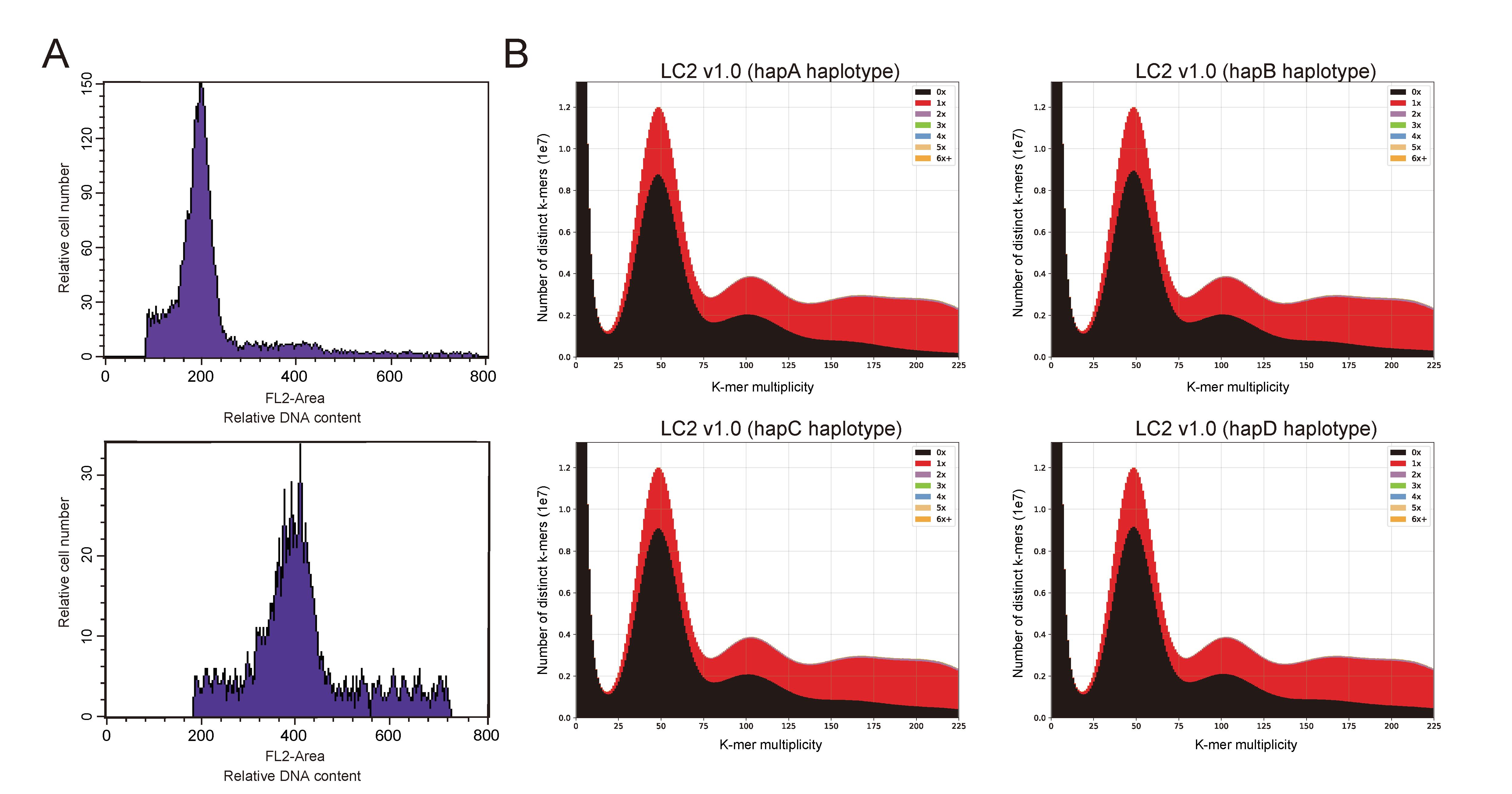
Figure S1.** Ploidy and phasing validation of *Actinidia arguta* cv. ‘Longcheng No.2’. (A) Ploidy analysis of *A. chinensis* diploid cv. ‘Hongyang’ (upper panel) and *A. arguta* tetraploid cv. ‘Longcheng No.2’ (lower panel) using flow cytometry. (B) Comparison of the amount of distinct K-mers absent and copy number variation between four haplotypes of LC2 v1.0 assembly and raw HiFi reads, respectively.



**Figure S2.** Expression patterns of loci with four allelic genes. (A) Expression levels of four-allele genes among homologous chromosomes. The expression level was presented in transcripts per kilobase per million mapped reads (TPM). (B) The expression patterns of inconsistent (upper panel) and consistent (lower panel) allelic specific expression genes (ASEGs) at different storage stages (1, 3, 7, 11 days after postharvest) in four haplotypes. D represent day(s) after postharvest.

**
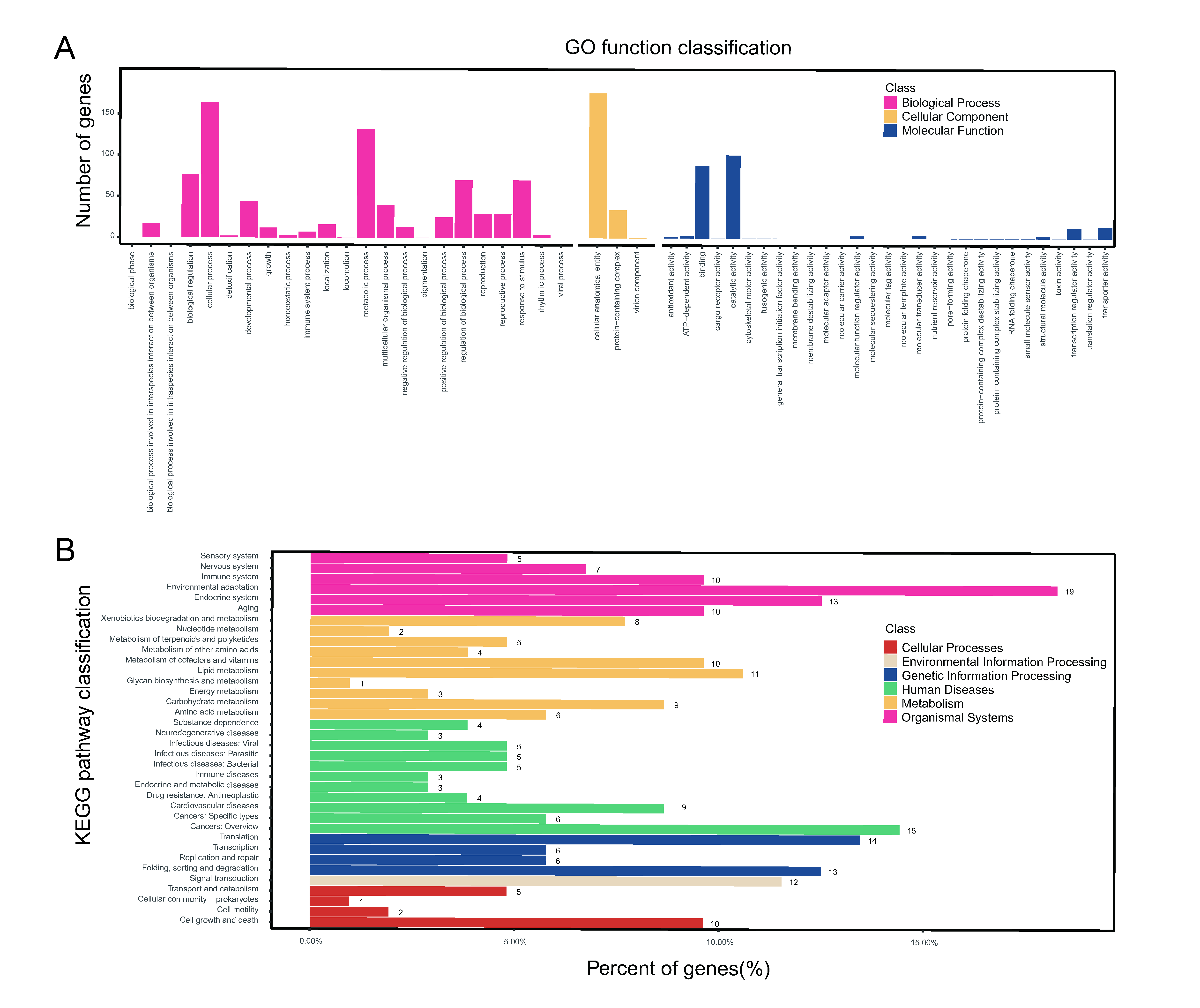
Figure S3.** GO and KEGG pathways enrichment analysis of 328 specific gene families in *Actinidia arguta* cv. ‘Longcheng No.2’. (A) GO functional classification of specific genes. (B) KEGG pathway classification of specific genes.


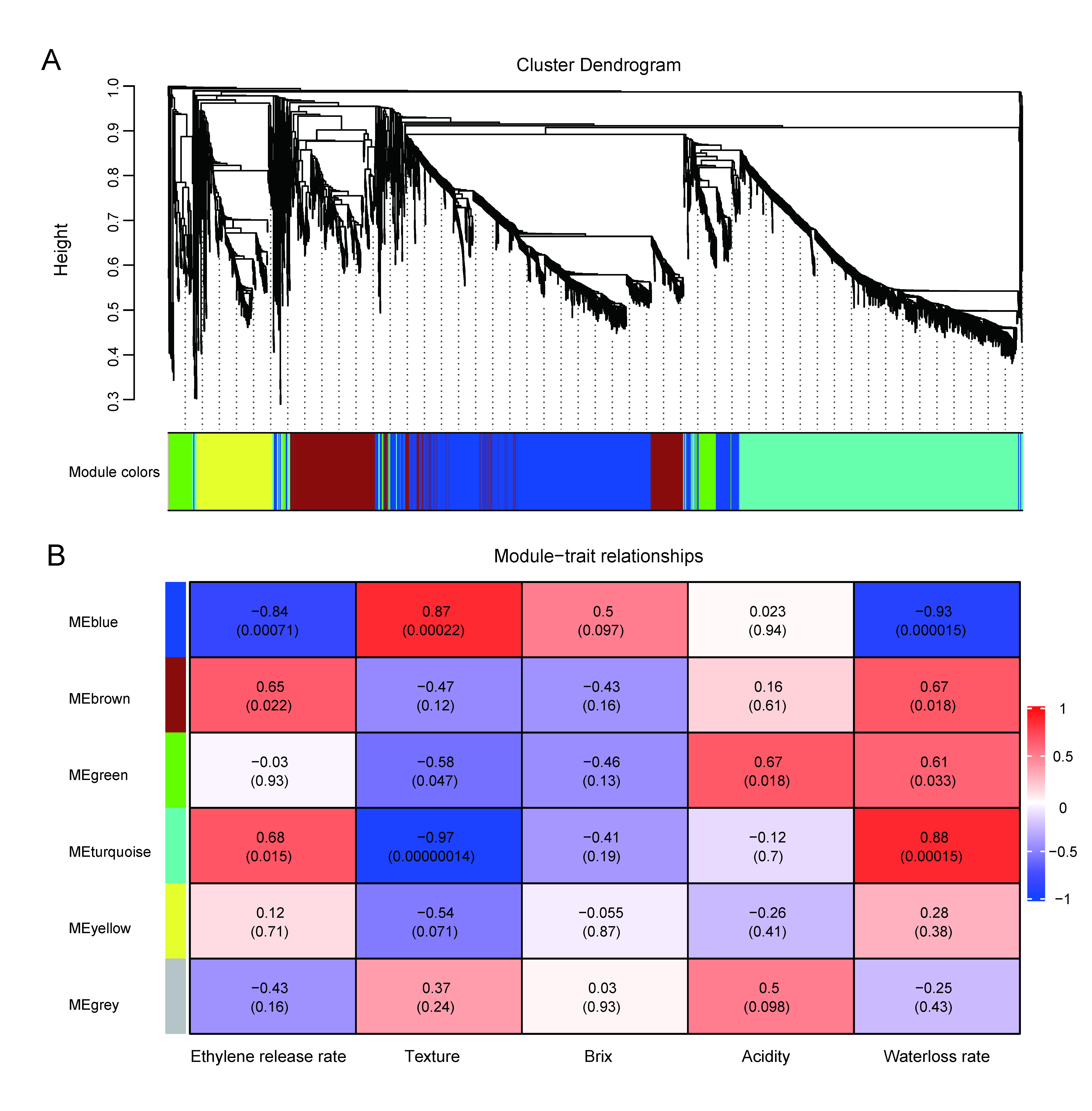
**Figure S4.** Identification of co-expression network modules in *Actinidia arguta* cv. ‘Longcheng No.2’. (A) Cluster dendrogram of genes subjected to any co-expression module. (B) Module-trait associations based on Pearson correlations. Red or blue color indicates a positive or negative correlation between the cluster and the trait, respectively.
